# Secondary Hemophagocytic Lymphohistiocytosis in a Patient With Crohn’s Disease Receiving Infliximab: A Diagnostic Challenge

**DOI:** 10.7759/cureus.95292

**Published:** 2025-10-24

**Authors:** Muneer Shoukath, Ananthu Sobhanan, Ajibola Omotosho, Nidhisha Kanakambujan, Mrinalini B Nandakumar, Bushra Jamil

**Affiliations:** 1 General Internal Medicine, North Manchester General Hospital, Manchester University NHS Foundation Trust, Manchester, GBR; 2 Medicine, The Aga Khan University and Hospital, Karachi, PAK

**Keywords:** biologic therapy, crohn’s disease, hemophagocytic lymphohistiocytosis, hlh, hyperferritinemia, hyperinflammation, immunosuppression, inflammatory bowel disease, infliximab, pancytopenia

## Abstract

Hemophagocytic lymphohistiocytosis (HLH) is a rare, life-threatening hyperinflammatory syndrome caused by uncontrolled immune activation. While pediatric HLH is well-recognized, adult cases often mimic sepsis or autoimmune flares, delaying diagnosis. Inflammatory bowel disease (IBD) and its immunosuppressive treatments, including infliximab, have been increasingly linked to HLH. We report a 65-year-old man with Crohn’s disease on infliximab who presented with persistent fever, chills, night sweats, headaches, and bradycardia. Laboratory testing revealed pancytopenia, hyperferritinemia (32,531 ng/mL), hypertriglyceridemia (11.5 mmol/L), hypofibrinogenemia (0.3 g/L), and elevated inflammatory markers. Infectious and autoimmune workups were negative, while CT imaging showed right upper lobe ground-glass opacities. Bone marrow biopsy demonstrated histiocytic hyperplasia with hemophagocytosis. The patient fulfilled HLH-2004 diagnostic criteria and had a high H-score. Infliximab was discontinued, and high-dose corticosteroids were initiated, resulting in marked clinical improvement without the need for etoposide. This case highlights infliximab-associated HLH in an IBD patient and emphasizes the importance of early diagnosis using established criteria. Clinicians should consider HLH in immunosuppressed patients presenting with unexplained systemic inflammation and cytopenias, as timely recognition and tailored therapy can significantly improve survival.

## Introduction

Hemophagocytic lymphohistiocytosis (HLH) is a rare, life-threatening syndrome characterized by excessive activation of macrophages and cytotoxic T-cells, leading to a hyperinflammatory state and multiorgan dysfunction [1,2]. It can be classified as primary (familial), resulting from genetic mutations affecting cytotoxic lymphocyte function, or secondary (acquired), often triggered by infections, malignancies, autoimmune diseases, or immunosuppressive therapies [1,2]. While HLH is well-recognized in pediatric populations, adult-onset HLH presents unique diagnostic challenges due to its nonspecific clinical manifestations, which often mimic sepsis, systemic inflammatory response syndrome, or autoimmune disease flares [3,4]. Prompt recognition is critical, as delayed diagnosis is associated with high mortality [3,4].

The diagnosis of HLH in adults relies on established criteria, including the HLH-2004 criteria and the H-score, both of which combine clinical, laboratory, and histopathological features to estimate the probability of HLH [3,4]. Typical findings include prolonged fever, cytopenias, hyperferritinemia, hypertriglyceridemia, hypofibrinogenemia, and evidence of hemophagocytosis in bone marrow or other tissues [3,4].

Inflammatory bowel disease (IBD), including Crohn’s disease, represents an emerging context in which secondary HLH can occur [5,6]. Chronic immune dysregulation inherent to IBD, along with immunosuppressive therapies such as corticosteroids, thiopurines, and biologics like infliximab, may predispose patients to HLH [5,6]. Viral infections, particularly with Epstein-Barr virus (EBV) or cytomegalovirus (CMV), can further trigger this hyperinflammatory response [5]. Although HLH is rare in IBD, case reports and series suggest that delayed recognition often results in poor outcomes, emphasizing the need for heightened clinical awareness [5,6].

Biologic therapies, particularly tumor necrosis factor-alpha (TNF-α) inhibitors like infliximab, have been implicated as potential triggers for HLH in IBD patients [5,6]. The TNF-α plays a critical role in immune regulation and cytotoxic lymphocyte activation; its inhibition may impair the clearance of infected or activated immune cells, creating a proinflammatory milieu conducive to HLH [5,6]. Additionally, TNF-α blockade may increase susceptibility to viral infections, which are well-recognized triggers of secondary HLH [5,6]. The combination of underlying immune dysregulation, immunosuppressive therapy, and infectious exposure may therefore synergistically precipitate HLH in susceptible individuals [5,6].

Here, we report a case of secondary HLH in a patient with Crohn’s disease receiving infliximab therapy, highlighting the diagnostic and therapeutic complexities in this population. By reviewing the literature and discussing clinical considerations, this report aims to improve recognition and management of HLH in adults with underlying chronic inflammatory conditions [1-7].

## Case presentation

A 65-year-old British man of Iraqi descent presented on 19th May 2025 with a four-day history of fever accompanied by rigors, night sweats, and fatigue. Two weeks prior, he had developed band-like headaches without associated nausea or vomiting. One week later, he experienced a transient loss of consciousness following a fall at work, witnessed by a customer. He also reported intermittent bradycardia (heart rate ~50 bpm) without associated symptoms.

**Figure 1:**
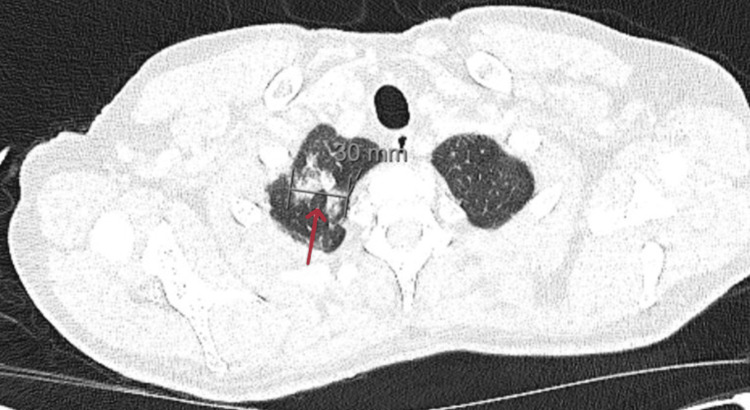
CT image showing focal ground glass changes right upper lobe in lung

His medical history included Crohn’s disease (diagnosed in 2021), iron deficiency anemia, asthma, hypercholesterolemia, cerebral atrophy, and colonic polyps. He had been receiving infliximab 120 mg subcutaneously every two weeks for six months, following intolerance to ustekinumab. Over the past year, he experienced a 6 kg unintentional weight loss. On admission, he was hemodynamically stable but febrile. Initial investigations (Table 1) revealed pancytopenia, elevated inflammatory markers (C-reactive protein (CRP) and erythrocyte sedimentation rate (ESR)), hyperferritinemia (32,531 ng/mL), hypertriglyceridemia (11.5 mmol/L), elevated lactate dehydrogenase (LDH) (539 IU/L), and hypofibrinogenemia (0.3 g/L). Cultures (blood, urine, respiratory) and imaging (chest X-ray, MRI brain) were unremarkable. A CT thorax-abdomen-pelvis demonstrated right upper lobe ground-glass opacities (Figure 1). 

**Table 1:** Hematological and biochemical parameters on presentation and after two months of treatment with reference range Hb: Hemoglobin, CRP: C-reactive protein, LDH: Lactate dehydrogenase

Blood test	On presentation	After two months of treatment	Reference range
WBC	0.7×10⁹/L	5.7×10⁹/L	4.0- 11.0 x 10^9/L
RBC	3.71×10¹²/L	4.56×10¹²/L	4.50-6.00x10^12/L
Hb	107 g/L	143 g/L	130.0 – 180.0 g/L
Platelets	52×10⁹/L	250×10⁹/L	150.0- 400.0 x 10^9/L
Neutrophils	0.26×10⁹/L	4.33×10⁹/L	1.80-7.50 x 10^9/L
Lymphocytes	0.42×10⁹/L	1.00×10⁹/L	1.00-4.00x10^9/L
Monocytes	0.10×10⁹/L	0.28×10⁹/L	0.20-1.00 x10^9/L
CRP	139 mg/L	<1 mg/L	0-5 mg/L
Ferritin	32,531 ng/mL	435 ng/mL	15-400 mcg/L
Triglycerides	11.5 mmol/L	4 mmol/L	0.00-1.6 mmol/L
LDH	539 IU/L	183 IU/L	139- 249 IU/L
Fibrinogen	0.3 g/L	2.0 g/L	1.7-4.2 g/L

Autoimmune markers (antinuclear antibody (ANA), double-stranded DNA (dsDNA), antineutrophil cytoplasmic antibodies (ANCA)) and viral serologies (HIV, cytomegalovirus (CMV), Epstein-Barr virus (EBV), SARS-CoV-2, tuberculosis (TB)) were negative. Complement C3 (serum) was elevated at 1.75 (reference range 0.75-1.65 g/L), and IgE (serum) was elevated at 466.0 (reference range 0.0-113.9 kU/L). An H-score indicated a high probability of HLH (Tables 2-3).

**Table 2:** The H-score parameters for HLH diagnosis The optimal cut-off for the H-score [4] parameters is 169 (accurately classifies 90% of patients). HLH: Hemophagocytic lymphohistiocytosis

Parameter	Value	Points
Ferritin, ng/mL (or µg/L)	<2,000	0
	2,000–6,000	+35
	>6,000	+50
Triglycerides, mg/dL (mmol/L)	<132.7 (<1.5)	0
	132.7–354 (1.5–4)	+44
	>354 (>4)	+64
Fibrinogen, mg/dL (g/L)	>250 (>2.5)	0
	≤250 (≤2.5)	+30
AST, U/L	<30	0
	≥30	+19
Hemophagocytosis on bone marrow aspirate	No	0
	Yes	+35
Temperature, °F (°C)	<101.1 (<38.4)	0
	101.1–102.9 (38.4–39.4)	+33
	>102.9 (>39.4)	+49
Organomegaly	No	0
	Hepatomegaly or splenomegaly	+23
	Hepatomegaly and splenomegaly	+38
Number of cytopenias	1 lineage	0
	2 lineages	+24
	3 lineages	+34
Known underlying immunosuppression (HIV positive or receiving long-term immunosuppressive therapy , eg- glucocorticoids)	No	0
	Yes	+18
	Optimal Cut off	169

**Table 3:** HLH probability for the patient using the H-score The H-score [4] of 250 raises a 99% probability of HLH. AST: Aspartate aminotransferase, HLH: Hemophagocytic lymphohistiocytosis

Parameter	Values	Score
Ferritin, ng/mL (or µg/L)	10,000	50
Triglycerides, mg/dL (mmol/L)	9.3	64
Fibrinogen, mg/dL (g/L)	3.2	0
AST, U/L	21	0
Hemophagocytosis on bone marrow aspirate	Yes	35
Temperature, °F (°C)	>39	49
Organomegaly	No	0
Number of cytopenias	3 lineages	34
Known underlying immunosuppression (HIV positive or receiving longterm immunosuppressive therapy; e.g., glucocorticoids)	Yes	18
	Total score	250

Bone marrow biopsy confirmed histiocytic hyperplasia with hemophagocytosis. Although efforts were made to retrieve imaging of the bone marrow biopsy, technical difficulties prevented access to the image from the hematology department. Genetic testing excluded primary HLH.

Treatment included high-dose corticosteroids, with initial intravenous methylprednisolone transitioned to oral prednisolone at 1 mg/kg (75 mg). A structured taper was implemented: weekly reductions (5 mg to 10 mg) down to 25 mg, followed by 5 mg weekly to 5 mg, and finally 5 mg on alternate days for two weeks before cessation. Tapering was guided by regular outpatient hematology reviews and serial blood monitoring, in line with up-to-date guidance and institutional best practice. Adjunct management included Pneumocystis jirovecii prophylaxis with cotrimoxazole 960 mg orally once daily, antiviral cover with acyclovir 200 mg orally four times daily, and antifungal prophylaxis with fluconazole 200 mg orally stat tapered to 100 mg daily for seven days. Vitamin D and calcium supplementation were provided, and infliximab was discontinued. The patient showed marked clinical improvement and remained clinically stable at two-month follow-up, with only mild steroid-related side effects. There were no relapses, emergency visits, or hospital readmissions during this period. The HLH has not recurred, and the gastroenterology team’s decision on restarting biologic therapy remains pending.

## Discussion

Hemophagocytic lymphohistiocytosis in adults remains a diagnostic and therapeutic challenge due to its nonspecific presentation and overlap with other hyperinflammatory conditions such as sepsis, macrophage activation syndrome, and severe autoimmune flares [1,2,8,9]. The syndrome is characterized by dysregulated immune activation, excessive cytokine release, and hemophagocytosis, often leading to multiorgan dysfunction if untreated.

Our patient exhibited several hallmark features of HLH, namely persistent fever, pancytopenia, hyperferritinemia, hypertriglyceridemia, and hypofibrinogenemia, thus meeting multiple HLH-2004 diagnostic criteria [3]. Although a markedly elevated ferritin level (>10,000 ng/mL) is highly suggestive of HLH, it is not diagnostic. Its specificity in adults is limited, as extreme hyperferritinemia may also occur in sepsis, liver failure, malignancy, and autoimmune disease; therefore, ferritin must be interpreted within the full clinical context alongside other diagnostic criteria [10]. The H-score, a validated probability tool for secondary HLH, further supported a high likelihood of the diagnosis and justified prompt initiation of treatment [4].

The role of infliximab as a possible trigger is particularly relevant. Infliximab, a chimeric monoclonal antibody targeting TNF-α, is widely used in Crohn’s disease and other autoimmune conditions. While effective, TNF-α blockade can impair immune surveillance, increasing susceptibility to opportunistic infections and paradoxical immune dysregulation [5]. Clear-cut reports of infliximab-induced HLH remain rare, with systematic reviews and national registry studies confirming its infrequent recognition despite the growing incidence of HLH overall [11-13]. The underlying mechanism is thought to involve impaired cytotoxic T-cell and NK-cell function, leading to uncontrolled macrophage activation [14].

Management of HLH depends on the underlying trigger, disease severity, and patient comorbidities. While the HLH-94 and HLH-2004 protocols recommend etoposide-based regimens for severe or familial disease [3], emerging evidence suggests that selected secondary or drug-induced HLH cases may respond to corticosteroids alone if recognized early [4,8]. Recent UK clinical guidance also emphasizes the importance of early recognition and stratification of suspected adult HLH cases to improve outcomes. In cases of steroid-refractory HLH, second-line treatment with anakinra (recombinant IL-1 receptor blocker) is indicated. In steroid/anakinra-refractory HLH, third-line treatment like IV immunoglobulin, cyclosporine, or etoposide should be considered in line with multidisciplinary team discussions [15]. In our patient, high-dose corticosteroids, discontinuation of infliximab, and supportive measures resulted in rapid clinical and biochemical improvement, avoiding the need for cytotoxic therapy.

This case emphasizes three key points: (1) HLH should be considered in immunosuppressed adults with unexplained systemic inflammation and cytopenias; (2) infliximab and other biologics may act as triggers even in the absence of infection or malignancy; and (3) early diagnosis and tailored therapy can lead to favorable outcomes in less fulminant cases. Increased awareness among clinicians, particularly in gastroenterology and rheumatology, is essential to improve survival rates.

## Conclusions

Hemophagocytic lymphohistiocytosis is a rare but serious hyperinflammatory syndrome requiring a high index of suspicion, especially in adults with autoimmune disease or on biologic therapy. In this case, infliximab in a patient with Crohn’s disease was considered a likely contributing factor, though other potential triggers cannot be fully excluded. Early diagnosis, prompt withdrawal of the suspected agent, and corticosteroid therapy led to complete recovery without cytotoxic drugs. This case adds to the limited literature describing infliximab-associated HLH, underlining the rarity of such reports and the need for continued vigilance. Awareness of HLH in immunosuppressed patients may improve survival outcomes.
